# Comparison of respiratory pathogen infections in hospitalized patients before and during the COVID-19 pandemic in Shanghai, China

**DOI:** 10.1128/spectrum.00435-25

**Published:** 2025-09-23

**Authors:** Xiaoxiao Li, Jie Wang, Junhua Dai, Fenfen Xiang, Zixi Chen, Mengzhe Zhang, Jiawen Qian, Rong Wu

**Affiliations:** 1 Laboratory Medicine Department, Putuo Hospital, Shanghai University of Traditional Chinese Medicine 66322, Shanghai, China; 2 Laboratory Medicine Department, Xundian County First People's Hospital, Kunming, China; University of Texas Southwestern Medical Center, Dallas, Texas, USA

**Keywords:** respiratory pathogens, COVID-19, acute respiratory infections, hospitalization

## Abstract

This study aimed to assess the impact of COVID-19 on the prevalence of respiratory pathogens among hospitalized patients with respiratory tract infections in Shanghai, China. Patients with acute respiratory infections (ARIs) hospitalized at Putuo Hospital, Shanghai University of Traditional Chinese Medicine from January 2017 to December 2022 were collected. Indirect immunofluorescence assay (IFA) was used to detect the IgM antibody of nine common pathogens, including *Chlamydia pneumoniae* (CP), *Mycoplasma pneumoniae* (MP), parainfluenza virus (PIV), respiratory syncytial virus (RSV), influenza virus A (FluA), influenza virus B (FluB), adenovirus (ADV), *Legionella pneumophila* (Lp), and *Coxiella burnetii* (Cb). A total of 24,933 eligible patients were enrolled. The overall detection rate of respiratory pathogens in the pre-outbreak group (2017–2019, 32.05%) was significantly higher than that in the outbreak group (2020–2022, 11.48%, *P* < 0.001). MP (16.03%), Lp (2.43%), FluB (1.73%), and PIV (1.64%) were the main pathogens. Positivity for MP, FluB, and PIV declined significantly, whereas the detection rate of Lp was increased (*P* < 0.05) during the 2020–2022 period compared with the 2017–2019 period. Children (73.21%) experienced a significantly higher infection rate than adolescents (65.35%), adults (25.20%), and older adults (15.40%). MP+PIV was the most common co-detection pattern. The detection of CP, MP, PIV, RSV, FluB, ADV, and Lp differed significantly between the two periods. From 2020 to 2022, the detection rates of CP, MP, PIV, RSV, FluB, and ADV decreased, whereas the detection of Lp increased. Knowledge of common pathogens’ dynamics could serve as a reference for further prevention and control of ARIs.

**IMPORTANCE:**

This study leverages the changes in the common respiratory spectrum pre-pandemic and during the COVID-19 pandemic in hospitalized patients in Shanghai. These data may serve as a scientific foundation for the prevention and management of ARIs. Doctors and policymakers should pay attention to the changes in the epidemic trends and types of respiratory pathogens and maintain monitoring of respiratory pathogens to better control the prevalence of respiratory pathogens.

## INTRODUCTION

Respiratory tract infections (RTIs) impose a significant health burden, contributing to morbidity and mortality risks across all age groups (1). RTIs are caused by a diversity of pathogens, including bacteria, viruses, and fungi (2). *Mycoplasma pneumoniae* (MP), respiratory syncytial virus (RSV), and influenza (Flu) are recognized as significant pathogens in RTI patients, and the prevalence of those pathogens varies by countries, regions, seasons, and periods (3, 4). Previous studies have shown that non-pharmaceutical interventions (NPIs), including wearing masks, hand hygiene, and social distancing, may have had a significant impact on the transmission of common respiratory pathogens during the COVID-19 pandemic (5–7). However, the changes in the spectrum of common respiratory pathogens before and during the COVID-19 pandemic among hospitalized patients in Shanghai remain unclear.

To comprehensively assess the infection rates of common respiratory pathogens before and during the COVID-19 pandemic, we retrospectively analyzed the results of respiratory pathogen detection among hospitalized patients from January 2017 to December 2022 using a multiple indirect immunofluorescence assay (IFA) kit. This study reveals dynamic variations before and after the outbreak of COVID-19, clarifies the impact of NPIs on non-COVID respiratory pathogens across all age groups, and provides valuable insights into the treatment and prevention of RTI.

## MATERIALS AND METHODS

### Study participants

From January 2017 to December 2022, patients with ARIs admitted to the Department of Respiratory Medicine and Pediatrics of Putuo Hospital, Shanghai, China, were enrolled, including cases of both upper and lower respiratory tract infections. Inclusion criteria for individuals were as follows: (i) at least one manifestation of acute infection (fever [≥37.5°C], chills, or abnormal white blood cell differential) and (ii) at least one of the listed respiratory tract clinical manifestations (rhinorrhea, cough, sputum, shortness of breath, lung auscultation abnormality, or chest pain). Finally, a total of 24,933 patients with results of multiple indirect immunofluorescence assay (IFA) testing were enrolled in this study. All of the enrolled patients were divided into four age groups: children (≤5 years), adolescents (6–17 years), adults (18–60 years), and older adults (>60 years). The period between January 2017 and December 2019 was classified as before the COVID-19 pandemic, whereas the period between January 2020 and December 2022 was classified as during the COVID-19 pandemic. All methods were performed in accordance with the relevant guidelines and regulations. This study was approved by the Ethics Committee of Putuo Hospital, Shanghai University of Traditional Chinese Medicine. As the retrospective analysis was based on anonymized data, the need for individual informed consent was waived by the Institutional Review Board of Shanghai Putuo District Central Hospital.

### Sample collection

Three milliliters of venous blood were drawn from each patient. The samples were centrifuged at 2,000 *× g* for 10 min at 4°C. The serum was separated and stored at −20°C until assayed with the Pneumoslide IgM test.

### Pneumoslide IgM test (Vircell, Granada, Spain)

Atypical pathogens and respiratory viruses, including *Chlamydia pneumoniae* (CP), *Mycoplasma pneumoniae* (MP), parainfluenza virus (PIV), respiratory syncytial virus (RSV), influenza virus A (FluA), influenza virus B (FluB), adenovirus (ADV), *Legionella pneumophila* (Lp), and *Coxiella burnetii* (Cb) in the serum were detected using the Pneumoslide IgM kit (Vircell, Granada, Spain) in accordance with the standard operating procedures. IFAs for all nine pathogens were performed uniformly for each enrolled patient during the acute phase of illness, based on the inclusion criteria of the study. Each slide has 10 wells, with each well containing one of the above pathogen antigens and a cell control. Serum samples were diluted 1:1 with phosphate-buffered saline (PBS) and treated with anti-human IgG sorbent. The sorbent-treated diluted serum was added to every well and incubated for 90 min at 37°C, and then, the slide was washed twice with PBS and dried. The fluorescent IgM secondary antibody was added to the wells and incubated at 37°C for 30 min. The slide was washed twice with PBS, and the fluorescent signal was detected under a fluorescence microscope (EUROStar III Plus).

Apple-green fluorescence was observed in the nucleus, cytoplasm, and/or periphery in 1%–15% of the cells for positive samples with ADV, FluA, FluB, RSV, or PIV (with colored syncytial cells observed simultaneously in PIV and RSV). All bacteria in the case of Lp, CP, or Cb exhibit apple-green fluorescence. Apple-green fluorescence was observed in the periphery of the cell for positive samples for MP. A negative sample showed no fluorescence for Lp, CP, and Cb, and a red cellular pattern for MP, ADV, FluA, FluB, RSV, and PIV (8).

### Statistical analysis

Excel 2010 and SPSS 22.0 statistical software were used for data processing and analysis. Bubble plots were created with the ggplot2 and reshape2 packages in R (version 4.1.2). The categorical variables were summarized as frequencies and proportions. Chi-square tests were used to compare the positive detection rates of various pathogens in the respiratory tract among different groups. The linear-by-linear association and gamma values were used to evaluate the trend in pathogen prevalence over 6 years, and a *P* value < 0.05 was considered statistically significant for all analyses.

## RESULTS

### Demographic characteristics

**Fig 1:**
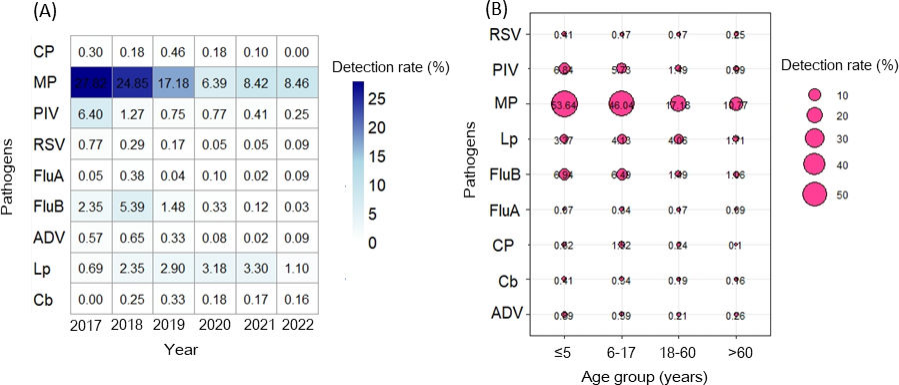
Heatmap showing the detection rates for nine respiratory pathogens by year (**A**); darker color representing higher rates. Bubble plot showing detection rates for nine respiratory pathogens by age group (**B**); bigger bubble size indicates higher detection rates.

In this study, we collected a total of 24,933 ARI cases from 2017 to 2022 in Shanghai, China, including 13,374 (53.64%) males and 11,559 (46.36%) females. The mean age of patients was 64.88 ± 25.49 years, with 59.99 ± 28.67 years for patients before the COVID-19 pandemic (2017–2019) and 70.86 ± 19.24 years for patients during the COVID-19 pandemic (2020–2022) (Table 1). The common respiratory pathogens were MP (16.03%), Lp (2.32%), FluB (1.73%), and PIV (1.64%). The detection rates of all pathogens significantly differed between years (*P* < 0.05) (Table 2). Changes in pathogen detection rates in each year are shown in Fig. 1A.

**TABLE 1:** Demographic and clinical characteristics of patients with respiratory infections from 2017 to 2022

Characteristic	Total (*n* = 24,933)	Pre-pandemic (2017–2019) (*n* = 13,725)	COVID-19 (2020–2022) (*n* = 11,208)	*χ* ^2^	*P*-value
Sex				16.28	<0.001
Male	13,374 (53.64%)	7,204 (52.49%)	6,170 (55.05%)		
Female	11,559 (46.36%)	6,521 (47.51%)	5,038 (44.95%)		
Age					<0.05
Mean (SD)	64.88 (25.49)	59.99 (28.67)	70.86 (19.24)		
Season				74.29	<0.001
Spring (3-5)	5,646	3,388 (24.68%)	2,258 (20.15%)		
Summer (6-8)	5,818	3,132 (22.82%)	2,686 (23.96%)		
Autumn (9-11)	6,453	3,416 (24.89%)	3,037 (27.10%)		
Winter (12-2)	7,016	3,789 (27.61%)	3,227 (28.79%)		
Pathogen					
CP	55	44 (0.32%)	11 (0.10%)	13.87	<0.001
MP	3,998	3,132 (22.82%)	866 (7.73%)	1043.89	<0.001
PIV	410	355 (2.59%)	55 (0.49%)	167.55	<0.001
RSV	60	53 (0.39%)	7 (0.06%)	26.93	<0.001
FluA	29	21 (0.15%)	8 (0.07%)	3.54	0.06
FluB	432	413 (3.01%)	19 (0.17%)	292.18	<0.001
ADV	76	69 (0.50%)	7 (0.06%)	39.35	<0.001
Lp	579	284 (2.07%)	295 (2.63%)	8.62	0.003
Cb	47	28 (0.20%)	19 (0.17%)	0.39	0.53
Total	5,686	4,399 (32.05%)	1,287 (11.48%)	1,482.64	<0.001

**TABLE 2:** Detection rates of respiratory pathogens during 2017–2022

Pathogen	Positive cases	Percentage for all sample (%)	95% CI for all samples (%)	2017 (*n* = 4,048)	2018 (*n* = 4,474)	2019 (*n* = 5,203)	2020 (*n* = 3,896)	2021 (*n* = 4,120)	2022 (*n* = 3,192)	χ^2^	P	Gamma value
CP	55	0.22	0.16-0.28	12 (0.30%)	8 (0.18%)	24 (0.46%)	7 (0.18%)	4 (0.10%)	0 (0.00%)	25.31	<0.001	−0.279
MP	3,998	16.03	15.58-16.49	1,126 (27.82%)	1,112 (24.85%)	894 (17.18%)	249 (6.39%)	347 (8.42%)	270 (8.46%)	1,263.43	<0.001	−0.390
PIV	410	1.64	1.49-1.80	259 (6.40%)	57 (1.27%)	39 (0.75%)	30 (0.77%)	17 (0.41%)	8 (0.25%)	690.57	<0.001	−0.667
RSV	60	0.24	0.18-0.30	31 (0.77%)	13 (0.29%)	9 (0.17%)	2 (0.05%)	2 (0.05%)	3 (0.09%)	62.97	<0.001	−0.586
FluA	29	0.12	0.07-0.16	2 (0.05%)	17 (0.38%)	2 (0.04%)	4 (0.10%)	1 (0.02%)	3 (0.09%)	34.25	<0.001	−0.036
FluB	432	1.73	1.57-1.89	95 (2.35%)	241 (5.39%)	77 (1.48%)	13 (0.33%)	5 (0.12%)	1 (0.03%)	523.64	<0.001	−0.573
ADV	76	0.30	0.24-0.37	23 (0.57%)	29 (0.65%)	17 (0.33%)	3 (0.08%)	1 (0.02%)	3 (0.09%)	48.67	<0.001	−0.506
Lp	579	2.32	2.13-2.51	28 (0.69%)	105 (2.35%)	151 (2.90%)	124 (3.18%)	136 (3.30%)	35 (1.10%)	106.43	<0.001	0.106
Cb	47	0.19	0.13-0.24	0 (0.00%)	11 (0.25%)	17 (0.33%)	7 (0.18%)	7 (0.17%)	5 (0.16%)	13.97	0.016	0.076

### Comparison of the detection rates of respiratory pathogens before and during the COVID-19 pandemic

Before the COVID-19 pandemic, the total detection rate of respiratory pathogens was 32.05% (2017–2019), and after the implementation of control measures due to the COVID-19 pandemic, the detection rate decreased to 11.48% (2020–2022) (*P* < 0.001). Overall, the detection rates of CP, MP, PIV, RSV, FluB, and ADV decreased during the COVID-19 pandemic, whereas that of Lp increased (*P* = 0.003). There were no significant differences in the detection rates of FluA and Cb (*P* > 0.05) (Table 1). More specifically, before the COVID-19 pandemic, the MP, FluB, and PIV ranked in the top three, with detection rates of 22.82%, 3.01%, and 2.59%, respectively. However, during the COVID-19 pandemic, the detection rate of FluB decreased significantly (*P* < 0.001). Lp replaced FluB and became the top two pathogens in the detection rate. Finally, the three pathogens with the highest detection rates were MP, Lp, and PIV, with 7.73%, 2.63%, and 0.49%, respectively (Table 2).

### Age-specific distribution and positivity rates

**Fig 2:**
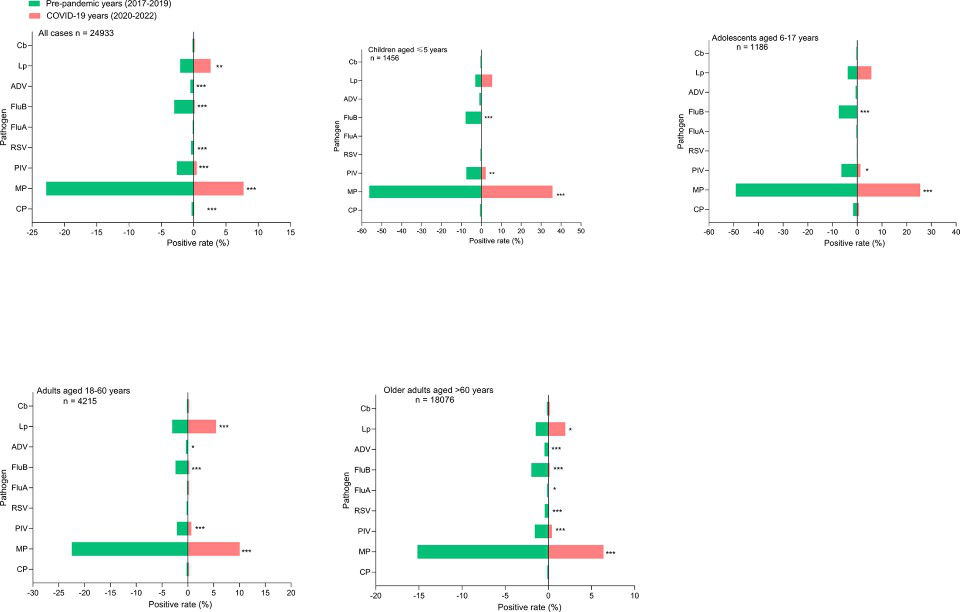
Comparison of the positive rates of nine respiratory pathogens in hospitalized patients among different age groups before (2017–2019) and during (2020–2022) the COVID-19 pandemic. *, *P* < 0.05; **, *P* < 0.01; and ***, *P* < 0.001.

Among patients with ARIs tested for all the nine pathogens, the highest rate of pathogen detection was seen in children (aged ≤5 years, 73.21% [1,066/1,456]), followed by 65.35% (775/1,186) in adolescents aged 6–17 years, 25.20% (1,062/4,215) in adults aged 18–60 years, 15.40% (2,783/18,076) in adults older than 60 years (Table 3). Changes in pathogen detection rates in each year group are shown in Fig. 1B. Positive detection rates in different age groups were compared between the pre-pandemic (2017–2019) and the pandemic (2020–2022) periods (Fig. 2). Before the COVID-19 pandemic, MP, FluB, and PIV ranked among the top three in the detection of pathogens in the children and adolescents groups. MP and Lp were the top two in the detection of pathogens in adults. During the COVID-19 pandemic, MP, Lp, and PIV were the top three in the detection of pathogens in all age groups (Fig. 2).

**TABLE 3:** Detection of respiratory pathogens in age groups

Pathogen	≤5 years (*n* = 1,456)	6–17 years (*n* = 1,186)	18–60 years (*n* = 4,215)	>60 years (*n* = 18,076)	*P*-value
CP	9 (0.62%)	18 (1.52%)	10 (0.24%)	18 (0.10%)	<0.001
MP	781 (53.64%)	546 (46.04%)	724 (17.18%)	1,947 (10.77%)	<0.001
PIV	100 (6.84%)	68 (5.73%)	63 (1.49%)	179 (0.99%)	<0.001
RSV	6 (0.41%)	2 (0.17%)	7 (0.17%)	45 (0.25%)	<0.001
FluA	1 (0.07%)	4 (0.34%)	7 (0.17%)	17 (0.09%)	<0.001
FluB	101 (6.94%)	77 (6.49%)	63 (1.49%)	191 (1.06%)	<0.001
ADV	13 (0.89%)	7 (0.59%)	9 (0.21%)	47 (0.26%)	<0.001
Lp	49 (3.37%)	49 (4.13%)	171 (4.06%)	310 (1.71%)	<0.001
Cb	6 (0.41%)	4 (0.34%)	8 (0.19%)	29 (0.16%)	<0.001
Total	1,066 (73.21%)	775 (65.35%)	1,062 (25.20%)	2,783 (15.40%)	<0.001

### Temporal distribution of respiratory pathogens

**Fig 3:**
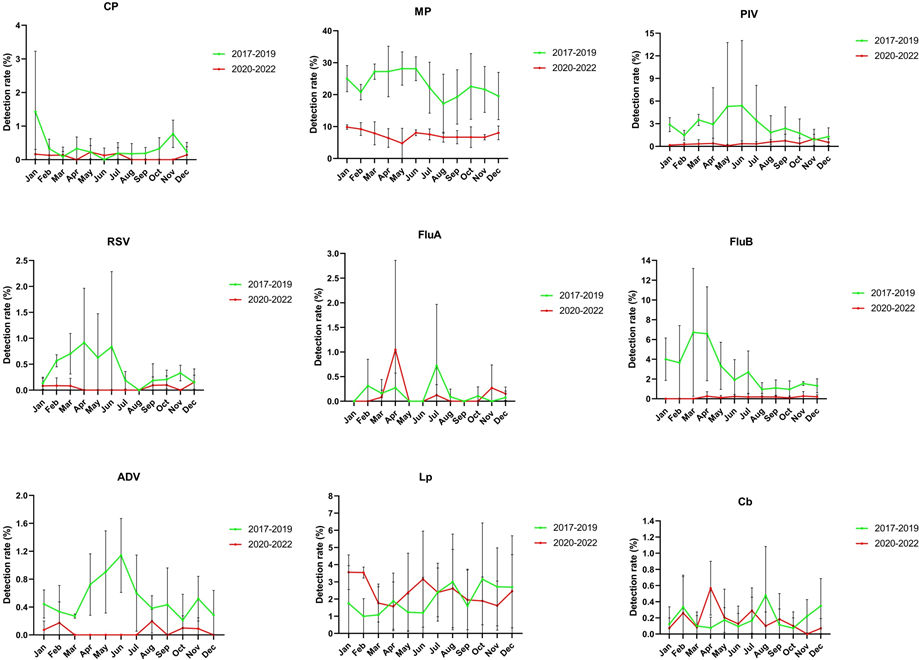
Comparison of the monthly distribution of each pathogen according to its detection rate in 2017–2022. The green line represents the average monthly detection rate from 2017 to 2019; the red line represents the average monthly detection rate from 2020 to 2022, and the vertical bar represents the standard deviation.

The monthly distributions of each pathogen before and during the COVID-19 pandemic are shown in Figure 3. From 2017 to 2019, CP largely occurred in January and November. The peak of the MP detection rate was between January and June, after which it declined sharply, reaching the lowest detection rate in August, then gradually increased. However, the detection rate of MP significantly decreased after the COVID-19 pandemic outbreak. A similar seasonal pattern was also observed in RSV. The average detection rate of PIV increased from February, reaching its highest in May and June. The peak month of the FluA detection rate shifted from July to April due to the COVID-19 pandemic. FluB mainly circulated in winter and spring, with the major peak between January and April. The average detection rate of ADV increased continuously from March to June and then gradually declined. Surprisingly, ADV was not detected between March and July from 2020 to 2022. The epidemic peak of Lp was from July to December. During 2020–2022, LP occurred in both hot and cold months, with a major peak in January to February and a minor peak in June to August. The overall monthly detection rate trend did not change.

### Co-detection pattern of pathogens

**Fig 4:**
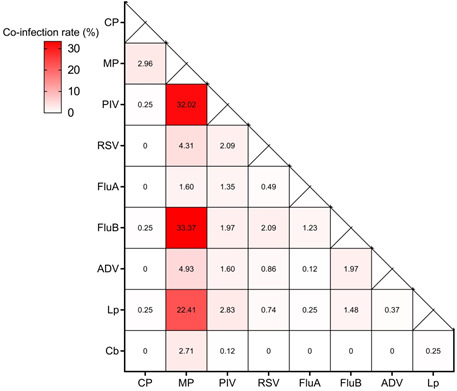
Mixed infection pattern of respiratory pathogens. The darker color of solid squares represents the higher frequency of the combination of these two pathogens. The proportion of co-infection was calculated by the count in mixed infection of each pair of co-infection/all counts of mixed infection.

Co-detections, in which more than one pathogen tested positive, were observed in 807 specimens, with a detection rate of 3.24% of all specimens. Among the 807 co-infection cases, double infections were identified in 726 (89.96%) cases, including 227 (28.13%) cases of MP + PIV, 226 (28.00%) cases of MP + FluB, and 154 (19.08%) cases of MP + Lp. Triple infections were detected in 71 (8.80%) cases, and quadruple infections were detected in 10 (1.24%) cases (Table 4). The commonly encountered co-infection patterns were MP and FluB, MP and PIV, and MP and Lp, as shown in Figure 4.

**TABLE 4:** Co-infections of respiratory pathogens in patients during 2020–2022 compared with 2017–2019

	2017–2019	2020–2022	Total
Double infections, *n* (%)			
MP + FluB	220 (27.26%)	6 (0.74%)	226 (28.00%)
MP + PIV	216 (26.77%)	11 (1.36%)	227 (28.13%)
MP + Lp	90 (11.15%)	64 (7.93%)	154 (19.08%)
MP + RSV	16 (1.98%)	1 (0.12%)	17 (2.11%)
Other	87 (10.78%)	15 (1.86%)	102 (12.64%)
Total	629 (77.94%)	97 (12.02%)	726 (89.96%)
Triple infections, *n* (%)			
MP + PIV + Lp	9 (1.12%)	1 (0.12%)	10 (1.24%)
MP + LP + FluB	9 (1.12%)	0	9 (1.12%)
MP + RSV + FluB	8 (0.99%)	0	8 (0.99%)
MP + ADV + FluB	6 (0.74%)	0	6 (0.74%)
Other	27 (3.35%)	11 (1.36%)	38 (4.71%)
Total	59 (7.31%)	12 (1.49%)	71 (8.80%)
Quadruple infection, *n* (%)			
MP + RSV + ADV + FluB	3 (0.37%)	1 (0.12%)	4 (0.50%)
MP + PIV + ADV + FluB	2 (0.25%)	0	2 (0.25%)
Other	4 (0.50%)	0	4 (0.50%)
Total	9 (1.11%)	1 (0.12%)	10 (1.24%)

## DISCUSSION

In this study, we analyzed the detection of respiratory pathogens in ARI among hospitalized patients in Shanghai from 2017 to 2022. The total detection rate was 22.81% in Shanghai, which was similar to that of a previous study in Gansu Province (29.2%) (9), and higher than that reported in Shenzhen (14.55%) (10), Beijing (5.64%) (11), and north China (7.6%) (12), but lower than that in Shaanxi Province (36.01%) (13), Shandong Province (35.75%) (14), and Xiamen (56.36%) (15). These differences may be affected by multiple factors, including geographic location, investigated period, climate conditions, study population, and methodological approaches to pathogen detection. Our study showed that the overall detection rates of respiratory pathogens were 32.05% in 2017–2019 and 11.48% in 2020–2022. The World Health Organization declared COVID-19 a public health emergency of international concern on January 30, 2020. During the COVID-19 pandemic, China initially implemented containment measures, followed by the adoption of a dynamic zero-case policy. A range of NPIs, including droplet and contact precautions (face mask use and increased hand hygiene), societal restrictions (school closures and reduced workplace attendance), isolation of infected individuals, and vaccination, were implemented to curb the spread of COVID-19, which greatly reduced the prevalence of the common respiratory pathogens. Furthermore, other confounding factors, such as reduced healthcare access and changes in patient populations, may also have contributed to the reduction in overall pathogen detection rates.

The results showed that MP was the predominant pathogen with the highest detection rate, followed by Lp, FluB, and PIV. Previous data also showed that MP was the most common atypical bacterium in other studies (16, 17). During the COVID-19 pandemic (2020–2022), a significant decrease in the detection rates of CP, MP, PIV, RSV, FluB, and ADV was observed in comparison to the pre-pandemic period (2017–2019). Interestingly, we also noticed a significant increase in the positive rate of Lp, indicating that not all pathogens were restricted by positive prevention (18). This may be explained by the fact that Lp infections are directly contracted from environmental sources and can be transmitted in healthcare or senior-living settings (19). Lp is mostly spread by inhaling infected aerosols or dust aspiration from contaminated soil. In general, it is accepted that water stagnation and poor maintenance of the water system in buildings are risk factors for Lp growth. In the context of the COVID-19 pandemic, many public health institutions have been severely affected by “stay-at-home” orders. All non-urgent hospital activities were suspended, and some wards were closed, with a consequent reduction in the use of the water system, the formation of stagnant water, and diminished disinfection in hospital water networks and cisterns. These conditions may have increased the risk of hospitalized patients’ exposure to waterborne pathogens, including Lp, thereby raising the Lp infection of patients (20). A previous study revealed that the hospital water network of the three examined wards closed for 3 months because of the COVID-19 emergency showed a higher Lp contamination after the lockdown period (21). Another study conducted in Spain has demonstrated that hotels that suffered the longest prolonged closures (>3 months) could have carried a higher risk of exposure to Lp in the domestic hot water system (22). Furthermore, the incidence of Lp has been high since the onset of the COVID-19 pandemic in Japan (19).

FluA and FluB are major contributors to seasonal epidemics. A recent study showed that NPIs had a strong suppressive effect on FluA and FluB, with the highest cumulative positivity rate of FluA + FluB in 2023 (31.9%) and the lowest rate in 2021 (2.0%) (23). In our study, the highest positivity rate of FluA + FluB was observed in 2018 (5.7%) and the lowest rate in 2022 (0.1%), which were significantly lower than those reported by the Chinese National Influenza Center (CNIC) and hospital-based data in Chengdu (23, 24). Moreover, it has been reported that FluA and FluB nearly disappeared during the COVID-19 phase among children in Guangzhou, China, consistent with our results (25). However, we should also note that pre-pandemic, pandemic, and post-pandemic comparative analysis could be influenced by multiple factors, such as changes in healthcare access (more severe hospitalized and tested patients) and diagnostic practices for pathogens in different phases.

It has been reported that children were notably susceptible to respiratory pathogens both before and during the COVID-19 pandemic (26). The reason might be explained by the lower innate immunity response of children than that of adults (27). Our results also showed that young children aged ≤5 years (73.21%) exhibited a significantly higher positive rate of common respiratory pathogen infections compared with adults (25.20%) and the elderly (15.40%), consistent with a recently published study (28). Therefore, it should be emphasized that young children are at higher risk of being infected with respiratory pathogens. During the COVID-19 pandemic (2020–2022), the detection rates of predominant pathogens among children in Shanghai were MP, Lp, and PIV. In comparison, in the pre-pandemic period (2017–2019), the top three detected pathogens were MP, FluB, and PIV. The pathogen spectrum of children has been impacted by the COVID-19 pandemic. In a national data covering the 2009–2019 period for children, RSV, FluA + B, human rhinovirus (HRV), MP, and PIV were the top five detected pathogens (29).

MP infections usually occur in winter and spring but can happen throughout the year, which was consistent with the seasonal pattern of MP during 2017–2019. Notably, the positive rate of MP markedly declined in 2020–2022 and showed no obvious seasonality. Additionally, an obvious detection peak of ADV was observed in the spring of the pre-pandemic. No detection peak was observed during the pandemic, with low incidence throughout the year. These findings were similar to the results reported by Xu et al. (30). The detection peak of PIV was observed in spring and summer in 2017–2019, which was consistent with seasonality reported in hospitalized children with lower respiratory tract infections (LRTIs) (31). However, PIV was detectable at a low rate throughout the year during the pandemic. Many studies have shown that NPIs associated with reduced transmission of COVID-19 have also reduced influenza (32, 33). Indeed, the common prevalent seasonal pathogens, such as RSV and Flu, with a few cases, were observed during COVID-19, and other studies also showed similar findings (34, 35). Lp infections were mainly observed in summer and autumn in Shaanxi Province, northwest China (13). We discovered that Lp was more common in the hot or cold seasons during COVID-19.

In this study, co-infections with at least two pathogens were only detected in 3.24% of the patients, which appears lower than those in some previous studies reported by Zhao et al. (8.5%, in Shijiazhuang, China, from January 2021 to December 2023 using multiplex PCR) (36) and Liu et al. (9.1%, Shaanxi province, from January 2014 to December 2018 using IFA) (13). This may be explained by variation in different diagnostic sensitivities, demographic characteristics, bacterial and viral types, different regions, and the investigated period. It has been reported that bacterial and viral co-infection may present more severe clinical outcomes (37), and MP + RSV was the most common type of viral-atypical bacterial co-infections (38). In this study, the most common combination was MP + PIV, which was consistent with that reported in Lanzhou, China (39). A previous study also indicated MP was the most frequently detected pathogen in co-infections with PIV type 3 (PIV3) (40). Due to the lack of information on its clinical severity, we are unable to determine the significance of co-infection in this study. Some studies have demonstrated that multiple infections were associated with prolonged hospital stay, admission to intensive care units, long-term mechanical ventilation support, and death (41).

There are some limitations to our study. First, this study was performed in a single center in a restricted geographic area, potentially impacting the generalizability of the findings to other populations or regions. Although the sample size was sufficient for a preliminary analysis, it may not have been large enough to detect minor effects or to fully explore the interactions between pathogens and demographic factors. Second, only the Pneumoslide IgM test was employed: factors such as disease progression, nutrition, and immune status can affect the production of antibodies, which may underestimate the positivity rate of certain pathogens. Moreover, the testing kit does not encompass all pathogens; it cannot cover all possible co-infecting viral, bacterial, or fungal respiratory pathogens. Our study did not include testing for emerging pathogens such as SARS-CoV-2, which limits the comprehensiveness of the results. Subsequent studies should broaden the detection spectrum of respiratory pathogens, enriching the epidemiological profile of ARIs. Third, the lack of comprehensive clinical data, including symptoms, laboratory results, and treatments, impeded the assessment of the association between these factors and pathogen positivity or disease severity. Finally, emphasizing the need for caution when extrapolating causality (e.g., NPI impacts) in this observational study, as other unanalyzed factors (such as environmental and health-seeking behavior changes, viral competition, or pathogen evolution) may have contributed to the observed shifts.

### Conclusion

In conclusion, this study showed that during the COVID-19 pandemic (2020–2022), the overall pathogen detection rate has significantly decreased, and the seasonal patterns of certain pathogens have also changed. An unknown number of variables, including NPIs, might be responsible for these changes. MP, Lp, and FluB were the most common respiratory pathogens, with children experiencing significantly higher infection rates. Strengthening vaccination coverage and implementing region-specific public health strategies that account for local environmental and social factors will be essential for mitigating the burden of respiratory pathogen infections in the post-pandemic period. Furthermore, hospitals and policymakers should continuously monitor the epidemiological and evolutionary dynamics of multiple respiratory pathogens to inform targeted intervention strategies and vaccination programs, thereby facilitating the effective management of acute respiratory infections.

## Data Availability

The original data that support the findings of this study are available from the corresponding author on reasonable request.
